# Sialic Acid—Modified Nanoparticles—New Approaches in the Glioma Management—Perspective Review

**DOI:** 10.3390/ijms22147494

**Published:** 2021-07-13

**Authors:** Przemyslaw Wielgat, Katarzyna Niemirowicz-Laskowska, Agnieszka Z. Wilczewska, Halina Car

**Affiliations:** 1 Department of Clinical Pharmacology, Medical University of Bialystok, Waszyngtona 15A, 15-274 Bialystok, Poland; hcar@umb.edu.pl; 2 Department of Experimental Pharmacology, Medical University of Bialystok, Szpitalna 37, 15-265 Bialystok, Poland; 3 Division of Chemistry, University of Bialystok, Ciołkowskiego 1K, 15-245 Bialystok, Poland; agawilczuwb@gmail.com

**Keywords:** sialic acid, nanoparticles, Siglecs, blood–brain barrier, glioma

## Abstract

The cell surface is covered by a dense and complex network of glycans attached to the membrane proteins and lipids. In gliomas, the aberrant sialylation, as the final stage of glycosylation, is an important regulatory mechanism of malignant cell behavior and correlates with worse prognosis. Better understanding of the role of sialylation in cellular and molecular processes opens a new way in the development of therapeutic tools for human brain tumors. According to the recent clinical observation, the cellular heterogeneity, activity of brain cancer stem cells (BCSCs), immune evasion, and function of the blood–brain barrier (BBB) are attractive targets for new therapeutic strategies. In this review, we summarize the importance of sialic acid-modified nanoparticles in brain tumor progression.

## 1. Introduction

Gliomas are a heterogeneous group of the most common and lethal primary brain tumors characterized by high histological variety and invading potential that underlie aggressive clinical course. The developmental diversity of brain tumors is closely related to the genetic and epigenetic alterations within the cancer genes that result in the vast cellular and molecular heterogeneity [1]. The changes in oncogenes (*EGFR*, *PDGF*) and suppressor genes (*TP53*, *p16^INK4a^*, *PTEN*) contribute to the genesis of high-grade gliomas and predict poor prognosis in age and sex-dependent manner [1,2]. Following the recent WHO classifications, the integration of molecular patterns and histological features highlights the possible therapeutic targets and improves the diagnosis and prediction [3,4]. However, the high biological activity of brain cancer stem cells (BCSCs) and separative function of the blood–brain barrier (BBB) are the main factors that promote glioma progression and attenuate the therapeutic effects of standard pharmacological therapies [5,6,7,8]. Since 2005, the temozolomide (TMZ)-based maintenance chemotherapy, besides surgical resection and radiation, is a standard of care in glioma management. Despite the high DNA alkylating efficacy of TMZ, the clinical benefits are not observed in at least 50% of TMZ-treated patients [9]. Therefore, targeting the BCSC therapeutic resistance, the development of new drug delivery systems, and effective therapeutic strategy in TMZ resistance is a major clinical challenge for high-grade gliomas treatment. The advances in the field of cancer biology highlighted the families of molecules that regulate the growth and invading potential of glioma cells. However, many smart targeting therapies to improve gliomas managements have failed due to the activation of multiple compensatory mechanisms, prohibited BBB crossing, and relatively low safety of drugs [1,10]. Therefore, small-molecule signaling inhibitors and tumor-specific monoclonal antibodies have been shown as insufficient to induce the complete malignancy regression [11]. In addition to the tumor cells’ originated molecules, the components of the microenvironment are an attractive target to develop better treatments for glioblastoma. The accumulating data have indicated that the interplay between tumor and inflammatory cells in the surrounding stroma leads to malignancy-promoting cell signaling with a focus on immune homeostasis [12]. The function of heterogeneous immune populations is manifested through the production and secretion of multiple mediators that regulate the activation or inhibition of the immune response. The cytokine secretory pattern of tumor-associated immune cells can be different in various cancers that suggest distinct scenarios by which immune control participates in malignancy progression [13]. In most cancers, the disruptions in the balance between opposite immune phenotypes are a part of the complex mechanism that supports cancer by damping the immune response [14]. In the context of gliomas, the immune compartment of the microenvironment, including resident microglia and infiltrating monocytes, macrophages or T cells, become dysfunctional through a molecular mimicry mechanism that changes their immune status from activation (M1) to immunosuppression (M2) [15]. The predominance of M2 cells and increased M2/M1 ratio has been shown to correlate with worse prognosis and shorter overall survival in patients with glioma [16,17]. This dependence is accompanied by the elevated expression of macrophage M2 phenotype markers, including CD163, as has been detected in both blood and tissue samples. The escape from the immune control is closely related to immune checkpoint pathways that are frequently exploited by tumor cells [18]. Upon the tumor cell surface ligand binding, the inhibitory immune receptors activate signaling pathways, leading to reduced immune cells activity in the field of cytokine production, phagocytosis, and proliferation. The accumulating data suggest that blocking the interactions between protein ligands and immune checkpoints using specific inhibitors and monoclonal antibodies allow the immune cells to become activated and destroy the tumor cells. Among the human immune checkpoints, the sialic acid–Siglec axis has been described as a new promising target in the immunotherapy of cancer [19,20]. The family of human Siglecs comprises 15 cell membrane receptors featured by the cell-specific distribution and recognition of sialoglycans. Differences in the structure of the intracellular domain of Siglecs determine the activating or suppressive signaling pathways responsible for the function of the immune cells. Most of the human CD33-related Siglecs recruit signaling pathways via immunoreceptor tyrosine-based inhibition motif (ITIM) and Src homology 2 domain-containing protein tyrosine phosphatase 1/2 (SHP-1/SHP-2) molecules that antagonize phagocytic processes [21,22,23]. Given the broad expression of sialic acids in malignancy and its correlation with cancer progression and immunity avoidance, it is believed that interactions between tumor sialic acid and Siglecs form the tumor immune microenvironment and weaken antitumor immunity [24]. Thus, by controlling the glycome in the tumor microenvironment and neutralization of Siglecs-dependent cellular activity, the efficacy of immune effectors against malignant cells could be potentiated in both cancer immunotargeting and conventional management. In addition to the biological functions, sialic acids modulate proteins, small molecule drugs, and carriers in drug delivery systems, resulting in pharmacokinetics improvement and toxicity reduction [25]. This review briefly focuses on the participation of sialic acid in nanocarrier-based brain tumor management.

## 2. Sialome as a Potential Target in Therapy of Glioma and Other Human Cancers

The engagement of sialic acids in cancer progression is closely related to their location in the glycan chains attached to the cell surface proteins and lipids that form dense and complex structures implicated in cell biology [26,27]. Structural studies of glycoconjugates have shown that most of the membrane glycosylated macromolecules bind sialic acids to the non-reducing end of the sugar chain, thus forming sialoglycans [28]. The main cellular function of sialic acids should be predominantly defined as the regulation of adhesive interactions that underlie the biological recognition in tissue homeostasis in the cell type-dependent manner. First, numerous sialylated glycoconjugates modulate homophilic adhesive interactions between cells of the single type and change tumor activity. Due to the terminal position of sialic acids residues, cell surfaces become the negative charge that induces attraction or repulsion between cell membrane molecules [29,30,31,32]. As a result, the malignancy-associated hypersialylation promotes the escape of cells from primary tumor mass and invasion of the surrounding area. The correlation between the density of cell membrane sialic acids and invasive potential has been found in various cancers. In the field of glioma immunity, the aberrant sialylation of the cell membrane glycocalyx is a crucial regulator of malignant cell behavior that correlates with worse prognosis and shorter patients’ overall survival [33,34,35]. Several sialoglycans, especially sialo–Lewis epitopes, through the interaction with selectins create the molecular basis of adhesion linked to the migration of cancer cells to the target organs through the vascular endothelium [36]. Therefore, Sialyl Lexis X (SLe^x^, CSLEX) has been described as a highly specific, sensitive, and prognostic marker in cancer diagnosis. The multidimensional analysis revealed that the overexpression of α2.8-sialic acid rich glycocalyx in highly malignant glioma cells promotes their migratory capacity and is a negative prediction marker compared to low-grade malignancies [37]. The reduction of aberrant sialylation has been also found as a powerful strategy in therapy of cancer. As shown, targeting sialome machinery with fluorinated sialic acid analogues reduces malignant cell activity in both cell culture and metastatic animal models [38]. In response to experimental exposure to fluorinated sialic acid analogue, P-3Fax-Neu5Ac, the depletion of α2,3- and α2,6-linked sialic acids was observed in cultured glioma GL261 cells as a result of α2,3- and α2,6-sialyltransferase inhibition that caused an impairment of interactions with extracellular matrix (ECM) components and migratory capacity. Similarly, the melanoma B16F10 cells treated with P-3F_ax_-Neu5Ac showed significantly reduced metastatic activity to the lungs and liver when transplanted in mice. Interestingly, these effects are enhanced due to nanoparticle-related inclusion and increased bioavailability [39,40]. In contrast to hypersialylation, the inhibition of sialylation in the most cancers improves their control by the immune system, as demonstrated by the increased level of CD^4+^ cells, CD^8+T^ cells, and NK cells [41,42].

In the second type of interactions, called heterophilic, sialic acids are involved in the cross-talk between malignant and the host cells of different histologic origin. In addition to the regulation of tumor cell phenotype, hypersialylation has been suggested to influence signaling pathways in the context of immunological synapses and thereby potentiates tumor immune evasion [5]. First, the aberrantly altered sialylation status closely correlates with the reduced cancer immunogenicity, which was described as a masking effect of cell surface antigens. Second, as mentioned previously, cell membrane sialic acids on tumor cells function as ligands that are recognized and bound to specific receptors. The recent studies highlight the involvement of the Siglec–sialic acid axis in the immune modulation; however, its therapeutic importance in cancer is still poorly developed [43]. The current trends in immunotherapy focus on the use of monoclonal antibodies that help turn the immune activity against cancer. This action is closely related to the modulation of the *“On”* and “Off” signaling system, which is functionally dependent on cell–cell and cell–matrix interactions [44]. Although the interplay between Siglecs and sialoglycans has been demonstrated in multiple malignant tissues, targeting this immune checkpoint is predominantly used in the management of leukemia [39]. Since elevated expressions of CD22 (Siglec-2) and CD33 (Siglec-3) are described as negative prognosis factors, the clinical use of anti-Siglec-2 and anti-Siglec-3 monoclonal antibodies conjugated with cytotoxins showed therapeutic benefits in patients with acute lymphoblastic leukemia (ALL) and acute myeloid leukemia (AML), respectively [45,46]. In addition, the small fragments of anti-Siglec antibodies exert high selectivity toward malignant, but not healthy, B cells when coupled with nanoparticles [47]. Recently, targeting Siglec-6 with specific antibody-conjugated chimeric antigen receptor T cells (Siglec-6-CAR-T cells) showed a morphologic complete response in a xenograft mouse model of chronic lymphocytic leukemia (CLL) [48]. In addition to the hematopoietic cancers, the strong effects of blocking antibodies against Siglec-7, Siglec-9, Siglec-10, and Siglec-15 have been demonstrated in cellular and animal models of cancers as inducers of NK cell-mediated cytotoxicity and macrophages-related phagocytosis [49]. Interestingly, in the breast tumor tissue, the activity of NK cells can be increased by the action of sialidases delivered as a complex with the targeting antibody and digesting the sialoglycans functionally bound to inhibitory Siglec-7 and Siglec-9 [39]. In the brain, sialoglycans are specifically recognized by Siglecs on the resident microglia and infiltrating peripheral immune cells, which leads to modulation of their defense function. Among highly sialylated brain glycoconjugates, the polysialylated neural cell adhesion molecules (PSA-NCAMs) are known as the only ligand for the Siglec-11 receptor in human microglia that participate in the signal transmission via inhibitory molecular switchers functionally linked with most of the CD33-related Siglecs [50]. In the case of the Siglec-11–PSA-NCAM axis, the bidirectional effects on the immune function of resident and infiltrating immune cells can be observed. First, the Siglec-related neuroprotection can result from restricted microglial sectretory activity and phagocytic capacity [51]. Second, the masking properties of sialoglycans expressed by the pathogens, cancers, and abnormal forms of biomolecules have been described in the neuropathology [52]. According to the recent hypothesis, glioma immune escape is facilitated by inhibitory CD33-related Siglecs that interact with sialic acid on malignant cells and trigger a reduced activation response, resulting in an improved survival of glioma cells [53,54,55,56]. The available clinical data from glioma patients showed enhanced Siglec expression profiles in both blood and the tumor niche, whereas their lower level in analyzed individuals was related to a better prognosis [19,53,57]. Interestingly, the Siglec-5 and Siglec-11 activatory counterparts, Siglec-14 and Siglec-16, respectively, deliver “eat me” signaling known to play a role in immune defense [58]. According to Virchof’s theory, inflammation is an inseparable feature of cancer progression. In the most cancers, the acute inflammation is a result of the host first line of defense; however, the immune evasion by malignant cells can lead to chronic phase that is highly attributed to malignancy [59]. In addition to the progression in tumor immunobiology, the role of inflammation and control mechanisms in glioma remains poorly understood compared to other types of cancers. Given the wide expression and importance of sialic acids in glioma biology, the sialome-related mechanism can be a candidate for targeting therapeutic strategies.

## 3. Theranostic Aspect of the Use of Nanoparticles (NPs) in Glioma

Recent decades indicated a strong concentration of scientific environment on the role of nanoparticles in the development of medical application including diagnostic and therapeutic approaches. It is established that nanotechnology creates a platform for a combination of diagnostics, therapeutics, and its delivery to the tumor with the subsequent monitoring of response [60]. These properties should be taken into consideration during the treatment of glioblastoma multiforme (GBM), which is a main malignant brain tumor. Moreover, it is one of the most challenging problems due to the fact that no currently available treatment is effective. To date, due to the unique physicochemical and biological properties, different nanomaterials including polymeric, liposome, metallic etc. have been engaged in the treatment of GBM [60,61]. However, apart from the EPR (enhanced permeability and retention) effect that allows nanotechnology to have an advantage over all other bioactive agents, to effectively gain the access to the GBM, active targeting to tumor tissue needs to be significantly improved via using specific homing ligands. In addition to the fact that NPs offers promising applications in cancer therapy and targeted drug delivery, more attention needs to be focused on the development of novel therapeutic approaches that will provide crossing the BBB, delivery of drugs to pathological areas of the brain with reduced side effects, and greater therapeutic efficiency.

### 3.1. Functionalization of Nanoparticles by Sialic Acid or Their Analogues Provides an Effective Way to Modulate Immune Response as Well as the Ability to Cross the Blood–Brain Barrier

**Figure 1:**
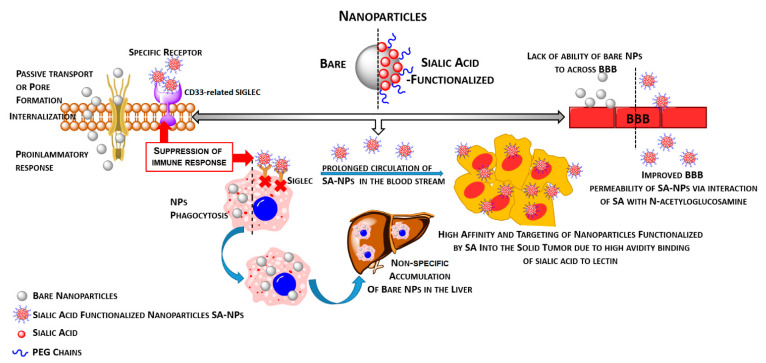
Modulation of the biological functions of nanoparticles after functionalization by sialic acid (SA). The SA-dependent chemical and physical features of NPs promote their activity in the field of immune response, BBB permeability, uptake and neutralization by phagocytes, and delivery to target malignant tissue.

Nanoparticles might interact with different components of the immune system, and depending on the intended use, NPs can either enhance or inhibit its function. So, their modulatory function can be useful or detrimental [62,63,64]. It is established that nanoparticles, especially unfunctionalized ones, possess the ability to generate the pro-inflammatory response. In comparison to functionalized NPs, they activate the macrophages and provide the secretion of pro-inflammatory cytokines such as IL-6 and TNF-α [65]. Moreover, the effect of nanoparticles on pro- or anti-inflammatory reaction also strongly depends on the dose, size, and surface modification of NPs [66]. Importantly, due to the small size, nanoparticles might internalize into cells via different approaches [67,68]. In the case of bare nanoparticles, they incorporate into the cells via passive targeting the formation of pores or mechanisms that engaged the endocytosis process; however, this process is non-specific and can not be adequately controlled [69,70]. Moreover, in the case of in vivo application, they can be captured by the RES (reticular endothelial system) and accumulate in critical organs such as liver and spleen [71]. On the other hands, among various types of synthesized nanomaterials, coating with hydrophilic polymers may protect against the undesirable interaction with the external environmental factors and in effect increase blood circulation times [69]. The ideal coating is a non-ionic hydrophilic flexible shell that prevents against opsonization, and as a consequence, restricts the NPs uptake by the phagocytic cells and extends the circulation half-life of the encapsulated drug [72]. Prolonged circulation times allow for passive targeting of the nanoparticles into tumors via the enhanced permeation and retention (EPR) effect or active targeting if homing ligands are engaged. In addition, during nanomaterial creation, the application of stimuli-responsive components (pH-, thermo-, light-, redox-, magnetic-sensitive) might allow releasing of the active agents only in the desired pathological site, thereby reducing the toxicity to healthy tissues [73,74]. In the field of nanotechnology, the application of sialic acids in the therapy of cancer has particular significance in both drug delivery and immunomodulation. First, the decoration of nanoparticles by sialic acids can enhance their delivery to tumor cells and therapeutic efficacy. The recent study by Xu et al. confirmed the strong apoptotic action of selenium nanocarriers against glioma cells [75]. However, their modification by sialic acid significantly increases the uptake by malignant cells and potentiates the apoptotic effect. Given this observation, the sialic acid-related activation of drugs could open new therapeutic strategies for highly resistant gliomas [76]. Second, the separative properties of the BBB as the main factor that limits therapeutic successes of glioma management can be modulated by sialic acids toward enhanced permeability. The BBB is made up of specialized vascular endothelial cells, which are characterized by an extremely low expression of leukocytes binding molecules as well as very tight junctions, which thereby results in the reduction of paracellular transport [77]. The main role of the BBB is associated with controlling the transport between the body fluid and the central nervous system, which is realized via various vesicular transporters at the apical membranes as well as via transcytosis only for lipophilic molecules with low molecular weights. Other molecules that do not fit the above listed criteria are fully rejected by the BBB [78,79]. Many reports indicated that bare and unmodified nanoparticles cannot effectively pass through the BBB [80,81,82]. However, results published by Kuo et al. indicated that effectively crossing the BBB and targeting BCSCs might be achieved after the specific modification of NPs [83]. The authors developed curcumin-loaded chitosan-poly(lactic-co-glycolic acid) NPs modified with sialic acid to permeate the BBB and with anti-aldehyde dehydrogenase (anti-ALDH) to target BCSCs. As shown in Figure 1, the crucial role of the sialic acid molecules is providing improvements in the permeability process due to interaction with N-acetyloglucosamine.

The modification of drug delivery systems by their sialylation improves the hydrophilic properties closely related to the sialic acid-dependent negative charge. As a result, the increased interaction potential is critical in crossing the BBB using a mechanism based on the endocytosis of sialoadhesins widely expressed in human brain microvascular endothelial cells (HBMECs) [83]. In line, the N-acetylglucosamine-rich HBMECs attract the highly negatively charged sialylated nanostructures and thus promote the BBB permeability [83]. Again, the sialic acid-determinated surface negative charge promote the avoidance of phagocytosis of nanocarriers by the mononuclear phagocytic system [65]. In contrast to positively charged biomaterials that undergo accumulation in the liver and spleen, the enhanced level of sialic acid-coated nanoparticles can be detected in the blood, thereby increasing their distribution and uptake by malignant cells due to overexpressed sialic acid recognizing lectins including galectins and selectins [65]. The increased permeability of the BBB is of particular importance in the context of pharmacological therapies of the brain pathologies. Tosi et al. demonstrated that loperamide, which is usually unable to cross the BBB, showed enhanced distribution within the brain when administered with sialic acid-coated nanoparticles [84].

Third, in the field of immunity, the sialic acid-coated nanocarriers can be engaged in immune receptors targeting to induce tolerance in the overactivated immune system or activate the defense mechanism in cancer-associated immune surveillance [39]. In the case of sepsis, NPs’ influence on immune cells activation provides the generation of an inflammatory response to infection that very often results in death. The application of sialic acid derivatives-functionalized NPs such as di(α2,8) N-acetylneuraminic acid (NANA), which suppresses the immune stimulation of macrophages and subsequently enables the nanoparticles to evade phagocytosis, might create new approaches in the anti-sepsis arsenal [65]. In both cellular and murine systemic models of sepsis, the treatment with modified nanocarriers alleviated inflammation through increased expression of interleukin-10 (IL-10) in macrophages. In the study by Spence et al., these effects were described as closely associated with an elevated expression of Siglec-E and inhibitory signaling as result of interaction with sialylated nanoparticles [85]. In contrast, there is increasing evidence that cancer progression is accompanied by the strong immune cell suppression due to the exposure of immune checkpoints on their ligands. The cancer-specific changes in sialylation are recognized by cell membrane-bound immune receptors as well as their soluble proteins and thereby actively contribute to cancer progression and immunity. In the brain, selectin P (SELP) is widely expressed in glioma cells and contributes to tumor progression closely associated with its adhesion-modulatory function, whereas the soluble form (sSELP) mediates the suppression of resident and infiltrating macrophages [86]. Hence, it has been described that multimeric forms of sulfated sialic acids are reactive with selectin-E and/or selectin-P and thus interfere with the interplay between selectins and their natural ligands [87]. Taken together, it could be concluded that published data suggest a critical role for the size and coating of nanomaterial in the biological interaction manner, while the application of a strong modulator such as sialic acid might open new ways for nanomaterials application.

### 3.2. Nanoparticle-Based Therapy and Sialic Acid–Siglec Interplay

**Figure 2:**
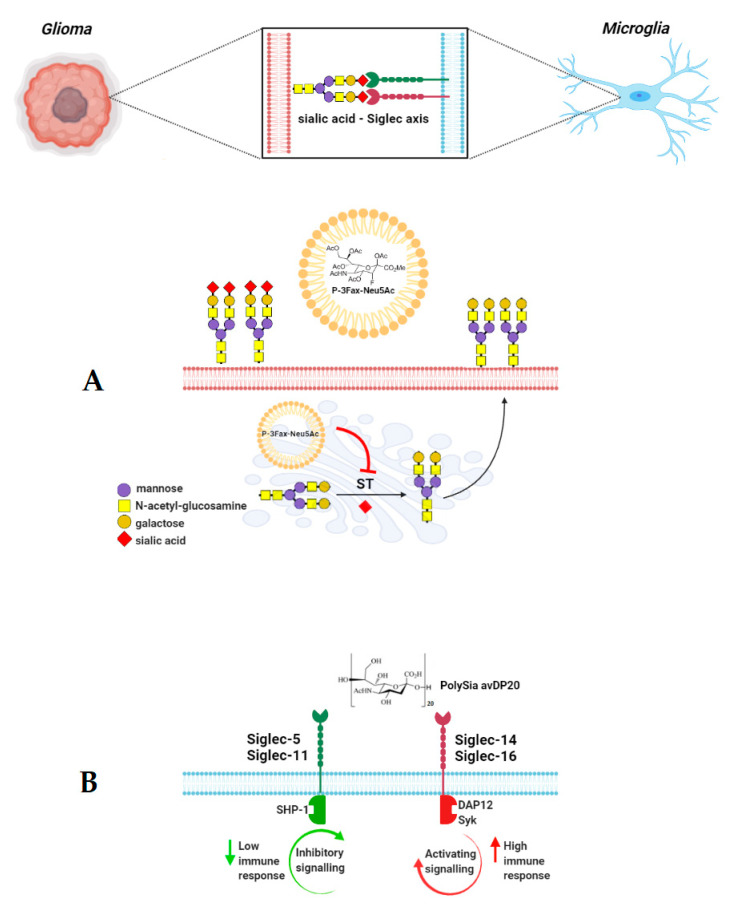
Possible strategies of targeting Siglecs and membrane sialome in glioma management. The administration of fluorinated sialic acid derivatives modulates the expression and activity of sialyltransferases (ST) in Golgi and results in the desialylation of the cell surface (**A**). Depending on inhibitory or activatory Siglec expression patterns, the binding of specific sialic acid polymers modulates the antitumor immune response (**B**). DAP12—AX activation protein of 12kDa; Syk—spleen tyrosine kinase; SHP-1—Src homology region 2 domain-containing phosphatase-1; SHP-2—Src homology region 2 domain-containing phosphatase-2.

Despite the promising therapeutic potential of nanoparticles, their clinical application has limitations in the context of uptake and clearance by the mononuclear phagocyte system, resulting in insufficient delivery to malignant cells. The current uptake decreasing strategy with polyethylene glycol-coated NPs (PEG-NPs) reduces the therapeutic value of these systems due to the internal production of PEG antibodies [88]. Among the regulators of evading phagocytosis, sialic acid is known as a “self” marker, which is recognized specifically by immunosuppressive glycan-binding receptors, especially monocytic inhibitory CD33-related Siglecs, including Siglec-5, Siglec-7, Siglec-9, Siglec-10, and Siglec-11. In this way, the interplay between sialic acid-covered nanoparticles and Siglec promotes the inhibition of phagocytic cells, including macrophages and microglia, and allows prolonged circulation in the bloodstream to the target tissue. This scenario has been confirmed by Kim et al. [65]. They showed that sialic acid-modified pegylated gold nanoparticles (sialic acid/PEG AuNPs) exhibited lower cellular uptake by macrophages when compared to unmodified neutral PEG AuNPs. This effect was accompanied by an enhanced distribution and the accumulation of sialic acid-modified NPs in tumor tissue [65]. In contrast, sialic acid-based nanotherapy can be also used as an immune activation-promoting strategy. In gliomas, tumor-infiltrating macrophages and microglia display mainly the M2 phenotype, which is known to promote malignant cell growth and survival, and microglia immunosuppression has been described as a negative prognostic marker in patients with glioblastoma multiforme [89,90]. The impaired immune function of the glioma microenvironment is attributed to the altered expression of genes and related proteins involved in the biological recognition processes. The cellular effects of sialic acid–Siglec interplay can be regulated by nanoformulated sialyltransferases inhibitors, e.g., fluorinated sialic acid derivatives, resulting in altered sialylation pattern or specific ligands characterized by binding capacity to inhibitory and/or activatory Siglecs. Given the importance of Siglecs in glioma biology, the activity of the tumor microenvironment can be reversed by targeting activatory receptors that counteract their paired inhibitory receptor. Human microglia express Siglec-11 and/or Siglec-16 that closely depend on the phenotype of the host. Siglec-11 and Siglec-16 are paired receptors characterized by 99% of sequence identity at the extracellular domain but opposite the intracellular signaling system based on ITIM and ITAM (immunoreceptor tyrosine-based activation motif), respectively [89,91]. Finally, both receptors have similar affinity to α2,8-linked sialic acids but activate opposite signal transduction systems. It has been shown that the low molecular weight polysialic acid with an average degree of polymerization of 20, called PolySia avDP20, prevents the activation of human macrophages and human microglia through the human lineage specific receptor Siglec-11 [91,92]. Sahraz et al. suggest that the PolySia avDP20-mediated anti-inflammatory effects might be a new therapeutic strategy in fibrillary amyloid-induced neurodegeneration [91]. If microglia-expressed Siglec-16 is functionally important for human immunity, the polymers of α2,8-linked sialic acids are potentially relevant in the host defense against glioma cells [90]. It is interesting, since Siglec-16 has been detected in glioma patients [19]. In line with this hypothesis, it is reasonable to target Siglec-16 using modified nanoparticles resulting in the activation of glioma-associated macrophages/microglia and counteracting the protective function of Siglec-11. Similarly, the local immune response can be also reversed by targeting the paired Siglec-5/Siglec-14 receptors expressed on cells in the glioma microenvironment in response to preferentially bound α2,3-sialoglycans (Figure 2A,B).

The functional importance of activatory Siglec-14 was confirmed in *SIGLEC-14^+/+^* individuals by elevated cytokines expression in macrophages compared to the cells with the lost *SIGLEC-14* gene [93]. Finally, new strategies were developed to interfere with the synthesis of sialoglycans in tumor cells and affect sialic acid dependent adhesion, migration, and viability. It has been demonstrated that targeting sialic acid molecules through the blockade of sialoglycans synthesis induces changes in the tumor immune microenvironment associated with pro-inflammatory effects and increased numbers of activated immune populations as well as the decreased modulatory action of Siglecs in the glioma microenvironment [94]. As suggested previously, sialic acid-blocking fluorinated derivatives exert strong effects against the tumor activity associated with aberrant sialylation. The intratumoral injection with P-3Fax-Neu5Ac and its intracellular delivery using a nanoparticle system prevents the incorporation of sialic acids by sialyltransferases widely expressed in the Golgi system [94,95]. The preclinical studies confirmed the safety of sialylation targeting strategy and its high efficacy in the range of metastasis inhibition [38].

**Figure 3:**
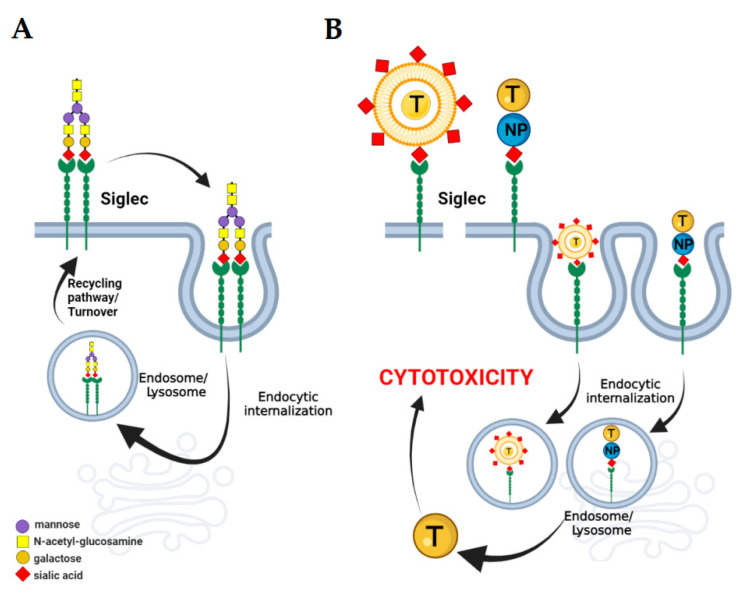
The endocytosis-based mechanism of action of sialic acid-decorated nanomaterials. Most Siglecs undergo endocytosis following binding with specific sialylated ligand. The endocytic uptake and recycling pathway control the structure of plasma membrane and related cellular activity (**A**). Nanomaterials functionalized with sialic acid can be conjugated with cytotoxin. Binding with the immune receptor is followed by endocytosis of the Siglec-recognizing complex. As a result of lysosomal degradation, the cytotoxin is released and induces a depletion of inhibitory/activatory Siglec-expressing immune cells (**B**).

In addition to the inhibitory and activating effects, Siglecs are recruited in the endocytic machinery of immune cells. Both CD22 and most of the CD33-related Siglecs undergo endocytic internalization that controls Siglec proteins turnover and underlies the host defense and pathogenicity in a clathrin/dynamin-dependent and independent manner, respectively. In addition, the endocytic capability can be exploited as a therapeutic target in the field of intracellular drug delivery [41,96]. Cell membrane Siglec proteins have been described to be recognized by specific antibodies and nanoparticles that exert an apoptotic effect when conjugated with cytotoxin (Figure 3).

In CD33-positive acute myeloid leukaemia (AML), leukemic blast cells are recognized by a specific anti-CD33 antibody that causes cytotoxicity due to its decoration with calicheamicins [97]. In line, the sialic-decorated nanostructures can act as alternative cytotoxic-coupled delivery systems. The endocytosis-based cytotoxic mechanism was described in human B-cell lymphoma cells exposed to CD22 ligands conjugated with saporin and auritoxin [98]. In the context of glioma therapy, targeting endocytic Siglecs can modulate the tumor environment by the depletion of suppressive immune cells and thereby reverse tumor immunity, as shown in multiple anticancer experimental managements [99].

## 4. Conclusions

The targeting molecular mechanisms underlying cellular adhesion and recognition is a promising therapeutic approach in the field of various cancers immunity. The involvement of sialic acid in basic cellular biological processes, such as adhesion, migration, differentiation, and recognition, suggest that sialoglycans are an attractive target and/or therapeutic tool for cancer. There are multiple limitations in a standard pharmacotherapy of various cancers, in particular, high-grade gliomas inspire developing new directions of targeted therapy. In the field of glioma management, the BBB permeability, drug distribution within the brain, and Siglec checkpoint functional importance might be the main goals of sialic acid-based therapy. Recent advances in nanotechnology suggest that sialic acid-modified nanoparticles present a promising strategy related to the mechanisms of brain tumor progression.

## Data Availability

Not applicable.
